# Exploring the long-term sequelae of childhood sexual abuse on risky sexual behavior among Chinese transgender women

**DOI:** 10.3389/fpsyg.2023.1057225

**Published:** 2023-04-14

**Authors:** Yingjie Chen, Ruijie Chang, Fan Hu, Chen Xu, Xiaoyue Yu, Shangbin Liu, Danni Xia, Hui Chen, Rongxi Wang, Yujie Liu, Xin Ge, Tiecheng Ma, Ying Wang, Yong Cai

**Affiliations:** ^1^School of Public Health, Shanghai Jiao Tong University School of Medicine, Shanghai, China; ^2^China Love Aid, Shenyang, China

**Keywords:** child sexual abuse, HIV/AIDS, multiple sexual partners, sexual behavior, transgender women, unprotected anal intercourse

## Abstract

**Introduction:**

Childhood sexual abuse (CSA) is a hidden but serious public health issue that can lead to a series of behavioral consequences and health problems in adulthood. It has been well documented that transgender women (TGW) have a high prevalence of CSA victimization. Moreover, risky sexual behaviors are also widespread among TGW; nevertheless, research investigating the associations between CSA victimization and risky sexual behaviors in TGW represents a gap in the literature.

**Methods:**

Our research was carried out mainly in Shenyang of China from November 2018 to January 2019. Sociodemographic characteristics, as well as information on participants’ HIV awareness and sexual behaviors, were collected through face-to-face interviews. The impact of CSA was examined through hierarchical logistic regression, adjusted for sociodemographic factors and HIV awareness.

**Results:**

In the sample of 247 adult TGW, 14.2% of them had a CSA history. In the previous 6 months, 30.8% of the participants reported condomless anal intercourse (CAI) and 38.5% of them had multiple sexual partners (MSP). The findings demonstrated that TGW with CSA history were more likely to take part in CAI (*p* = 0.001, OR = 4.252) or have MSP (*p* = 0.004, OR = 3.260) in adulthood. Furthermore, HIV knowledge was not a predictor of CAI or MSP, but higher HIV risk perception was associated with a greater probability of CAI.

**Conclusion:**

Transgender women with a history of CSA were more prone to engage in CAI and have MSP in China.

## Introduction

Childhood sexual abuse (CSA) is a public health concern worldwide, with high prevalence rates varying by geography, gender, or diverse method used for measurement (Baytunca et al., 2017). The definition of CSA given by the World Health Organization (WHO) includes any sexual exploitation that causes actual or potential harm to individuals under the age of 18 (WHO, 1999). Any forced or coerced sexual contact, not just penetration, implies the occurrence of sexual abuse (Sikkema et al., 2009). A meta-analysis from China indicated that the prevalence of CSA was 13.8% in boys and 15.3% in girls (Ji et al., 2013), and those among sexual minorities have been frequently much higher. In 2015, Xu et al. (2018) informed that 40.7% of participants self-reported contact or non-contact CSA in the investigation among men who have sex with men (MSM) from 30 provinces in China. It has been demonstrated that the rate of CSA exceeded 20% based on a cohort study including MSM in three Chinese cities (Li et al., 2021). The results from a regional sample in Guangzhou showed that nearly 30% of MSM have experienced CSA (Jiang et al., 2020). In other parts of the globe, a study conducted in New York City showed that the CSA rate among Latino MSM was over 20% (Levine et al., 2018), and a cohort study found that 35.5% of gay and bisexual men were victims of CSA (Lenderking et al., 1997). Furthermore, transgender women (TGW), whose gender identity is inconsistent with their male sex assigned at birth, living on the margins of society, are more victims of CSA than cisgender women (Sizemore et al., 2021; Friedman Burley et al., 2022).

Several studies have shown that the behavioral symptoms related to CSA, as well as adverse physical problems, can last until adulthood (Rooney et al., 2018), with HIV infection among the most severe (Brennan et al., 2007; Sikkema et al., 2009). When compared to samples from the other populations, TGW bear over 30 times the lifetime HIV infection risk than the general people (Reback and Fletcher, 2014), two times the HIV infection risk than MSM (UNAIDS, 2022), and it has been cited that the risky sexual behaviors, including condomless anal intercourse (CAI) and having multiple sexual partners (MSP), are the primary driver of HIV infection in TGW (Herbst et al., 2008; Baral et al., 2013). MSP lead to a high risk of acquiring HIV through increasing sexual activities and infection sources, while CAI may give rise to HIV infection by impairing the integrity of the fragile rectal mucosal epithelial barrier and inducing a local inflammatory response (Campbell et al., 2013; Cheng et al., 2014).

It has been demonstrated that a variety of factors are linked to risky sexual behaviors. Sexual behaviors vary with age, education level, marital status, and self-reported sexual orientation (Holmes, 2005; Castro, 2016; De Santis et al., 2017), and Hoyos (2001) also found the effect of income on consistent condom use. In addition, the role of HIV knowledge and/or perceived HIV risk, categorized as HIV awareness in this study, in determining sexual behaviors has been supported by several studies (Noroozinejad et al., 2013). A study of Mexican youth found associations between HIV knowledge and condom use (Tapia-Aguirre et al., 2004), and a meta-analysis from the United States reported a negative correlation between HIV-perceived risk and risky sexual behaviors (Marks et al., 2005). In addition, Noroozinejad et al. (2013) have shown the modifying effect of perceived HIV risk on the relationship between HIV knowledge and sexual risk behaviors among injecting drug users. However, the precise relevance of HIV awareness in achieving effective control over risky sexual behaviors remains controversial in the studies conducted in other populations (Gerrard et al., 1996; Tapia-Aguirre et al., 2004; Hall et al., 2008; Noroozinejad et al., 2013; Castro, 2016).

The influence of CSA on risky behaviors has also been widely explored. Among heterosexual men, those with a CSA history are more likely to have MSP than others (Schraufnagel et al., 2010). Bensley et al. (2000) observed an association between CSA history and CAI, and MSP in women (Holmes, 2005). Moreover, the long-term sequelae of CSA vary by gender; women with CSA history have a higher risk of engaging in risky sexual behavior than man (Abajobir et al., 2017). The aforementioned risk behaviors, having multiple sexual partners and condomless intercourse, prevail among TGW as well (Mullings et al., 2000; Homma et al., 2012). However, in many studies related to CSA impact, TGW have been neglected or excluded from enrollment, which means that reliable data from this population are scarce, and this is especially true in China (Rooney et al., 2018; Tang et al., 2018).

To promote equal health rights for people of all gender identities, studies on CSA impact are needed among this population. TGW are perceived as a population bearing high risk for both HIV and CSA. Given the high rates of HIV infection among survivors of CSA (Sizemore et al., 2021), we hypothesized that Chinese TGW with CSA experience are more likely to engage in risky sexual behaviors, and the factors of sociodemography and HIV awareness should be also taken into consideration. Our study aims to evaluate experiences of CSA victimization among Chinese TGW and investigate the association between CSA and the occurrence of risky sexual behaviors in adulthood in this population. We hope to bring a decrease to the HIV epidemic by reducing the underlying behavioral determinants and providing insights into HIV prevention strategies tailored for TGW in China.

## Methods

### Participants and recruitment

Recruitment was carried out from November 2018 to January 2019 in the cities of Shenyang and Kunming, China. Both cities are economically developed in the region and diversely populated, with large expatriate populations and high social inclusiveness, thus, they are more suitable for TGW to live in (Liu, 2019). Participants who met the following criteria were enrolled in our study: 1. age ≥ 18; 2. gender assigned at birth was male; 3. self-identified as female; 4. had anal sex with men within the past 6 months. Individuals who were unable to independently provide informed consent because of severe mental disorders or who were unable to provide a phone number for verification were excluded.

Collaboration has been established with a non-governmental organization (NGO) devoted to improving the physical and physiological health of TGW, to help us engage with the transgender community. In the initial stages of recruitment, five eligible individuals with large social networks in each city were collected as “seeds”; then, new participants got involved through referrals or invitations from the “seeds.” This form of recruitment, known as snowball sampling, was conducted in the aforementioned approach until reaching saturation, that is to say, there was no other individual who could be recommended for our study. An interviewer-administered questionnaire was utilized to gather information from eligible participants under the guidance of trained NGO staff, and the involved participants would receive 200 CNY as a reward.

### Ethics review

The investigation was approved by the Ethics Committee of the School of Public Health at Shanghai Jiao Tong University, China. Written informed consent was obtained from all subjects, and they reserved the right to withdraw at any time without being questioned.

### Measures

#### Sociodemographic characteristics

Participants were asked to fulfill the baseline survey, including age, education level, marital status, monthly income, and sexual orientation.

#### HIV awareness

##### HIV-KQ-18

HIV knowledge questionnaire (HIV-KQ-18) was used to assess knowledge about HIV transmission, diagnosis, and prevention. The tool has demonstrated good internal consistency, stability, and suitability for use even with low-literacy populations (Carey and Schroder, 2002; Johnston et al., 2011). It contains 18 questions with response options of true, false, and do not know (Carey and Schroder, 2002). The answer key “do not know” responses are also scored incorrectly. 1 point for correct, 0 point for incorrect, and the total score of the questionnaire was considered as an independent variable. The score ranged from 0 to 18, and higher scores indicate more knowledge about HIV.

##### HIV risk perception

Regarding the perception of HIV risk acquisition, in other words, the probability to acquire HIV according to a layperson’s understanding (Tugume et al., 2019), a self-composed scale comprising four items was used to measure the perceived risk of HIV infection of participants, with five available responses representing the extent from favorable to unfavorable, and the scores represented range from 1 to 5. The total score ranged from 4 to 20, and a higher total score implies that lower HIV risk is perceived (Supplementary material).

#### Childhood sexual abuse history

During the interview, each participant was asked “Have you been engaged in any sexual practice under inducement or coercion from others when you were under 18?,” responses were coded as yes or no. All coercive sexual experiences reported by the participants were defined as childhood sexual abuse (Levine et al., 2018).

#### Risky sexual behaviors

Risky sexual behaviors were documented by asking the participants about their history of anal intercourse in the previous 6 months. An affirmative response to the aforementioned question was followed up with inquiries about condom usage and the number of sex partners.

##### Condomless anal intercourse

The condom usage was obtained across frequency rating items asking “In the past 6 months, how often did you use condoms during anal sex with your male sexual partner?.” The answer options scale ranged from 1 (never) to 5 (constantly), since the condom usage data formed highly skewed and zero-inflated distributions, a dichotomous variable was created, and those who self-reported cannot always use condoms consistently (who did not respond with “5 (constantly)”) in anal sex with sexual partners were set as CAI (Wang et al., 2017).

##### Multiple sexual partners

In the past 6 months, participants who had both regular and irregular sexual partners/commercial sexual partners were described as having MSP. This risky sexual outcome variable was coded affirmatively if the participant acknowledged the engagement in any anal sex with a regular sexual partner and irregular/commercial sexual partners in the previous 6 months.

### Statistical analysis

Initial descriptive statistics on TGW were taken for all and stratified by the presence or absence of childhood sexual abuse experience. Kolmogorov–Smirnov test (K-S test) was used to evaluate the distribution of continuous variables, for data in the normal distribution, the mean ± standard deviation (SD) were used for description, and for non-normally distributed data, the central tendency was shown by median and quartiles. The chi-square test or rank-sum test was used to test for differences between groups. We then included the variable of HIV awareness among the participants, including the total score of HIV knowledge and HIV risk perception. Furthermore, we utilized the hierarchical logistic regression to assess the independent effect of childhood sexual abuse on risky sexual behaviors in adulthood.

Predicting variables were entered into the model in three blocks, with age, education level, monthly income, marital status, and self-reported sexual orientation entered in block one for the sake of adjusting sociodemographic characteristics. The score of KQ-18 and HIV risk perception entered into block two, and the history of CSA victimization was included in block three. The receiver operating characteristic (ROC) curves were computed to exhibit the performance of each model, and the area under the ROC curve (AUC) was calculated simultaneously using R packages “pROC” (Robin et al., 2011) for comparison among models. Statistical significance was assessed using an alpha value of 0.05 in two-tailed tests. All analyzes were conducted in R 4.0.5.

## Results

### Sample characteristics

The characteristics of the sample are summarized in Table 1. A total of 247 Chinese TGW enrolled in our study, and the age range of the respondents was 18–61 years old. This sample involves TGW from low-to-high education levels, and most participants had lower middle incomes, with monthly revenue lower than ¥12,000 accounting for 92.7%. More than half of the participants (56.3%) self-reported being homosexuals, and over 80% reported being single as their marital status.

**Table 1:** Characteristics of transgender women by childhood sexual abuse in China.

Independent variables	All		Childhood sexual abuse		No childhood sexual abuse		*P*-value
	*N*	%	*N*	%	*n*	%	
Total	247	100	35	14.2	212	85.8	
Sociodemographic characteristics							
Age group							
<30	102	41.3	10	28.6	92	43.4	0.256
30–45	122	49.4	21	60	101	47.6	
>45	23	9.3	4	11.4	19	9	
Education							
Primary school or less	20	8.1	3	8.6	17	8	0.820*
Middle school	70	28.3	12	34.3	58	27.4	
High school	80	32.4	11	31.4	69	32.5	
(including secondary school, vocational school)							
College and above	77	31.2	9	25.7	68	32.1	
Monthly income(CNY^a^)							
≤3,000	72	29.1	13	37.1	59	27.8	0.164
3,001–6,000	105	42.5	11	31.4	94	44.3	
6,001–12,000	52	21.1	6	17.2	46	21.7	
>12,000	18	7.3	5	14.3	13	6.2	
Marital status							
Single and will not marry the opposite sex	165	66.8	22	62.9	143	67.4	0.244*
Single and will marry the opposite sex	36	14.6	6	17.1	30	14.2	
Married heterosexual women	10	4	0	0	10	4.7	
In a marriage of convenience	7	2.8	0	0	7	3.3	
Divorced or widowed	29	11.8	7	20	22	10.4	
Self-reported sexual orientation							
Heterosexual	37	15	7	20	30	14.1	0.469*
Homosexual	139	56.3	21	60	118	55.7	
Bisexual	44	17.8	6	17.1	38	17.9	
Pansexual	13	5.3	1	2.9	12	5.7	
Unsure	14	5.6	0	0	14	6.6	
HIV awareness							
HIV-KQ 18	12.00(10.00,15.00)**		11.94 ± 3.22**		12.00(10.00,15.00)**		0.943
HIV risk perception	10.00(8.00,13.00)**		10.86 ± 2.76**		10.00(8.00,13.00)**		0.553
High risk sexual behaviors							
CAI							
Yes	76	30.8	19	54.3	57	26.9	0.001
No	171	69.2	16	45.7	155	73.1	
MSP							
Yes	95	38.5	22	62.9	73	34.4	0.001
No	152	61.5	13	37.1	139	65.6	

Unless indicated otherwise, data are given as *n* (%), statistically significant results are bolded in the table, CNY–Chinese yuan, USD–United States dollar, HIV-KQ-18–HIV knowledge questionnaire 18 items, CAI—condomless anal intercourse, MSP–multiple sexual partners, INR–interquartile range.

*Fisher’s exact test, **continuous type variables are represented by mean ± SD or median(INR).

^a^CNY 3,000 equivalent to 466 USD; 6,000 equivalent to 931 USD.

The objectives were divided into two groups based on whether they had experienced CSA, and the CSA group accounted for 14.2% of the total. There was no statistical difference between groups for the aforementioned sociodemographic characteristics. All of them were placed into Model 1, the results demonstrated the correlation between MSP and self-reported sexual orientation, and MSP or CAI was not related to any other sociodemographic variables.

### HIV awareness

Internal consistency of the total HIV-KQ-18 score demonstrated acceptable reliability (Cronbach’s *α* = 0.785), and similar performance also appeared in the HIV risk perception scale (Cronbach’s *α* = 0.701). The results of the K-S test demonstrated that the score of HIV-KQ-18 and HIV risk perception scale does not follow the normal distribution (*P*<0.001). The median score was 12.00 for the HIV-KQ-18 and 10.00 for the HIV risk perception scale (see Table 1). Variables related to HIV awareness were entered into Model 2, and no association was found between HIV knowledge and MSP or CAI (see Tables 2, 3). Furthermore, the result referred that higher levels of HIV risk perception were accompanied by high rates of CAI (*P*<0.001, OR = 0.820; see Table 2); however, no such correlation was shown when we examined MSP (see Table 3).

**Table 2:** Hierarchical logistic regression models of condomless anal intercourse.

Variables	Model 1					Model 2					Model 3				
	*B*	*P*	OR	95%CI		*B*	*P*	OR	95%CI		*B*	*P*	OR	95%CI	
				Lower	Upper				Lower	Upper				Lower	Upper
Block 1 demographic characteristics															
Age group															
<30	REF	REF	REF	REF	REF	REF	REF	REF	REF	REF	REF	REF	REF	REF	REF
30–45	0.576	0.093	1.779	0.908	3.485	0.610	0.092	1.841	0.906	3.742	0.494	0.188	1.639	0.786	3.418
>45	0.065	0.909	1.068	0.350	3.257	−0.036	0.951	0.964	0.305	3.052	−0.174	0.772	0.840	0.259	2.726
Education level															
Primary School or less	REF	REF	REF	REF	REF	REF	REF	REF	REF	REF	REF	REF	REF	REF	REF
Middle School	−0.564	0.314	0.569	0.190	1.704	−0.674	0.259	0.510	0.158	1.641	−0.813	0.188	0.444	0.132	1.489
High school	−0.775	0.188	0.461	0.145	1.460	−0.918	0.142	0.399	0.117	1.361	−1.068	0.098	0.344	0.097	1.218
(Including secondary school, vocational school)															
College and above	−1.158	0.062	0.314	0.093	1.058	−1.024	0.122	0.359	0.098	1.313	−1.197	0.080	0.302	0.079	1.152
Marital status															
Single and will not marry the opposite sex	REF	REF	REF	REF	REF	REF	REF	REF	REF	REF	REF	REF	REF	REF	REF
Single and will marry the opposite sex	−0.125	0.790	0.883	0.353	2.210	−0.110	0.822	0.896	0.343	2.341	−0.210	0.678	0.811	0.301	2.182
Married heterosexual women	0.127	0.866	1.135	0.261	4.937	−0.137	0.863	0.872	0.186	4.092	0.069	0.931	1.071	0.226	5.077
In a marriage of convenience	1.031	0.227	2.803	0.527	14.897	1.247	0.182	3.480	0.558	21.695	1.427	0.133	4.166	0.647	26.838
Divorced or widowed	1.022	0.028	2.778	1.119	6.901	0.895	0.070	2.448	0.931	6.436	0.817	0.102	2.264	0.849	6.035
Monthly income															
≤3,000	REF	REF	REF	REF	REF	REF	REF	REF	REF	REF	REF	REF	REF	REF	REF
3,001–6,000	−0.412	0.251	0.663	0.328	1.338	−0.454	0.224	0.635	0.306	1.320	−0.393	0.308	0.675	0.317	1.436
6,001–12,000	−0.698	0.119	0.497	0.207	1.198	−0.688	0.140	0.503	0.201	1.254	−0.672	0.160	0.511	0.200	1.304
>12,000	−0.894	0.217	0.409	0.099	1.691	−1.041	0.170	0.353	0.080	1.563	−1.284	0.103	0.277	0.059	1.297
Self-reported sexual orientation															
Heterosexual	REF	REF	REF	REF	REF	REF	REF	REF	REF	REF	REF	REF	REF	REF	REF
Homosexual	0.263	0.561	1.301	0.536	3.158	0.312	0.511	1.366	0.539	3.459	0.348	0.479	1.417	0.540	3.716
Bisexual	−0.277	0.618	0.758	0.255	2.253	−0.131	0.822	0.877	0.279	2.755	−0.110	0.856	0.896	0.273	2.940
Pan sexual	0.975	0.225	2.652	0.548	12.826	1.445	0.091	4.244	0.794	22.688	1.704	0.051	5.494	0.993	30.404
Unsure	0.231	0.763	1.260	0.280	5.680	0.162	0.835	1.176	0.254	5.442	0.412	0.607	1.510	0.315	7.247
Block 2 HIV awareness															
HIV-KQ18						−0.037	0.446	0.963	0.875	1.061	−0.041	0.416	0.960	0.869	1.060
HIV risk perception						**−0.198**	**0.001**	**0.820**	**0.741**	**0.907**	**−0.221**	**0.001**	**0.802**	**0.720**	**0.893**
Block 3 childhood sexual abuse											**1.447**	**0.001**	**4.252**	**1.812**	**9.977**
Nagel kerke *R*^2^	0.140					0.226					0.279				
△*R*^2^	0.140					0.086					0.053				

Statistically significant results are bolded in the table, OR–odds ratio, REF–reference group, HIV-KQ-18–HIV knowledge questionnaire 18 items.

**Table 3:** Hierarchical logistic regression models of multiple sexual partners.

Variables	Model 1					Model 2					Model 3				
	*B*	*P*	OR	95%CI		*B*	*P*	OR	95%CI		*B*	*P*	OR	95%CI	
				Lower	Upper				Lower	Upper				Lower	Upper
Block 1 demographic characteristics															
Age group															
<30	REF	REF	REF	REF	REF	REF	REF	REF	REF	REF	REF	REF	REF	REF	REF
30–45	0.625	0.050	1.868	1.001	3.484	0.617	0.053	1.854	0.991	3.467	0.547	0.094	1.728	0.910	3.281
>45	−0.237	0.675	0.789	0.261	2.390	−0.231	0.683	0.794	0.262	2.406	−0.299	0.603	0.742	0.240	2.290
Education level															
Primary School or less	REF	REF	REF	REF	REF	REF	REF	REF	REF	REF	REF	REF	REF	REF	REF
Middle School	−0.434	0.444	0.648	0.213	1.968	−0.462	0.421	0.630	0.204	1.940	−0.513	0.381	0.599	0.190	1.888
High school	−0.956	0.108	0.385	0.120	1.233	−0.972	0.104	0.379	0.117	1.221	−1.035	0.089	0.355	0.108	1.172
(Including secondary school, vocational school)															
College and above	−0.547	0.368	0.579	0.176	1.903	−0.597	0.338	0.550	0.162	1.865	−0.661	0.296	0.516	0.149	1.784
Marital status															
Single and will not marry the opposite sex	REF	REF	REF	REF	REF	REF	REF	REF	REF	REF	REF	REF	REF	REF	REF
Single and will marry the opposite sex	0.611	0.136	1.842	0.825	4.113	0.625	0.130	1.868	0.832	4.192	0.583	0.168	1.792	0.783	4.104
Married heterosexual women	0.952	0.175	2.592	0.655	10.249	0.967	0.170	2.629	0.661	10.456	1.108	0.116	3.029	0.762	12.046
In a marriage of convenience	−20.909	0.999	0.000	0.000		−20.855	0.999	0.000	0.000		−20.704	0.999	0.000	0.000	
Divorced or widowed	0.474	0.305	1.606	0.649	3.972	0.481	0.300	1.618	0.651	4.017	0.346	0.463	1.414	0.561	3.563
Monthly income															
≤3,000	REF	REF	REF	REF	REF	REF	REF	REF	REF	REF	REF	REF	REF	REF	REF
3,001–6,000	0.682	0.061	1.978	0.968	4.039	0.688	0.060	1.989	0.973	4.067	0.795	0.034	2.215	1.063	4.616
6,001–12,000	0.273	0.528	1.314	0.562	3.072	0.265	0.542	1.303	0.556	3.052	0.323	0.470	1.381	0.575	3.320
>12,000	1.417	0.025	4.125	1.193	14.268	1.428	0.024	4.172	1.202	14.480	1.352	0.042	3.865	1.052	14.205
Self-reported sexual orientation															
Heterosexual	REF	REF	REF	REF	REF	REF	REF	REF	REF	REF	REF	REF	REF	REF	REF
Homosexual	1.050	0.020	2.858	1.183	6.903	1.062	0.019	2.891	1.193	7.008	1.107	0.017	3.024	1.214	7.533
Bisexual	0.317	0.552	1.373	0.484	3.898	0.324	0.545	1.383	0.484	3.951	0.390	0.478	1.477	0.503	4.334
Pan sexual	−0.013	0.988	0.987	0.195	4.998	−0.041	0.961	0.960	0.188	4.911	0.071	0.934	1.073	0.201	5.722
Unsure	0.882	0.237	2.417	0.560	10.425	0.902	0.228	2.464	0.569	10.667	1.106	0.147	3.023	0.679	13.456
Block 2 HIV awareness															
HIV-KQ18						0.017	0.715	1.017	0.930	1.112	0.017	0.710	1.017	0.929	1.115
HIV risk perception						0.005	0.903	1.005	0.926	1.091	0.001	0.976	1.001	0.921	1.089
Block 3 childhood sexual abuse											**1.182**	**0.004**	**3.260**	**1.464**	**7.260**
Nagel kerke *R*^2^	0.157					0.158					0.200				
△*R*^2^	0.157					0.001					0.042				

Statistically significant results are bolded in the table, OR–odds ratio, REF–reference group, HIV-KQ-18–HIV knowledge questionnaire 18 items.

### Risky sexual behaviors

#### Childhood sexual abuse history as a predictor of condomless anal intercourse

**Figure 1:**
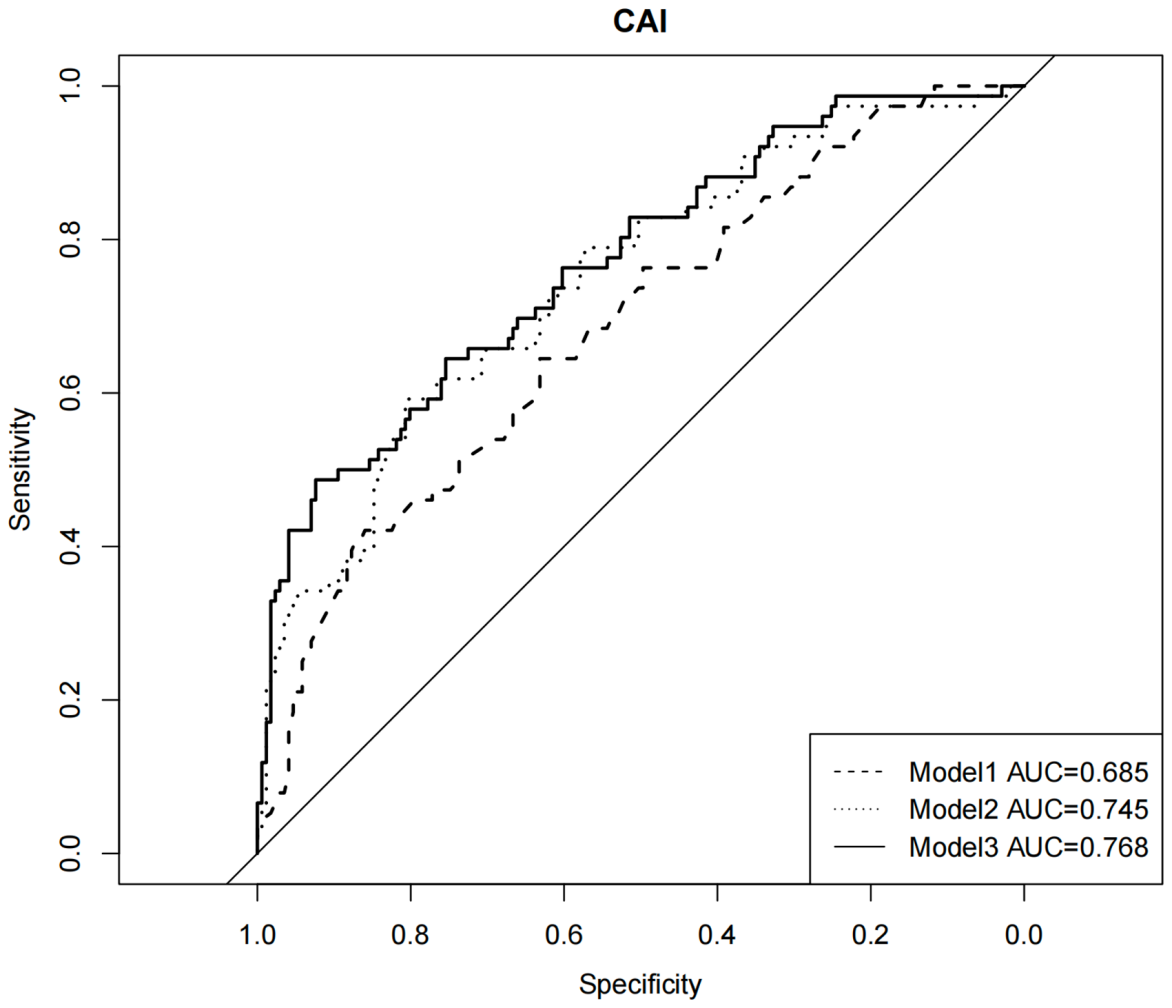
Receiver operator characteristic curve for demographic factors, HIV awareness variables, child sexual abuse in hierarchical logistic regression models predicting condomless anal intercourse (*N*=247).

Overall, 30.8% of the sample reported CAI in the past 6 months are listed in Table 1. Lower scores on the HIV risk perception scale indicated an increasing HIV risk perception with a slight incremental probability of CAI (*P*<0.001, OR = 0.802; see Table 2). The rate of CAI exposure was significantly higher in participants with CSA than without CSA (*p* = 0.001, OR = 4.252). As depicted in Figure 1, three constructed models for CAI showed better fit with the addition of new variables, and the area under the ROC curve increased from 0.685 in Model 1 to 0.745 in Model 2 to 0.768 in Model 3.

#### Childhood sexual abuse history as a predictor of multiple sexual partners

**Figure 2:**
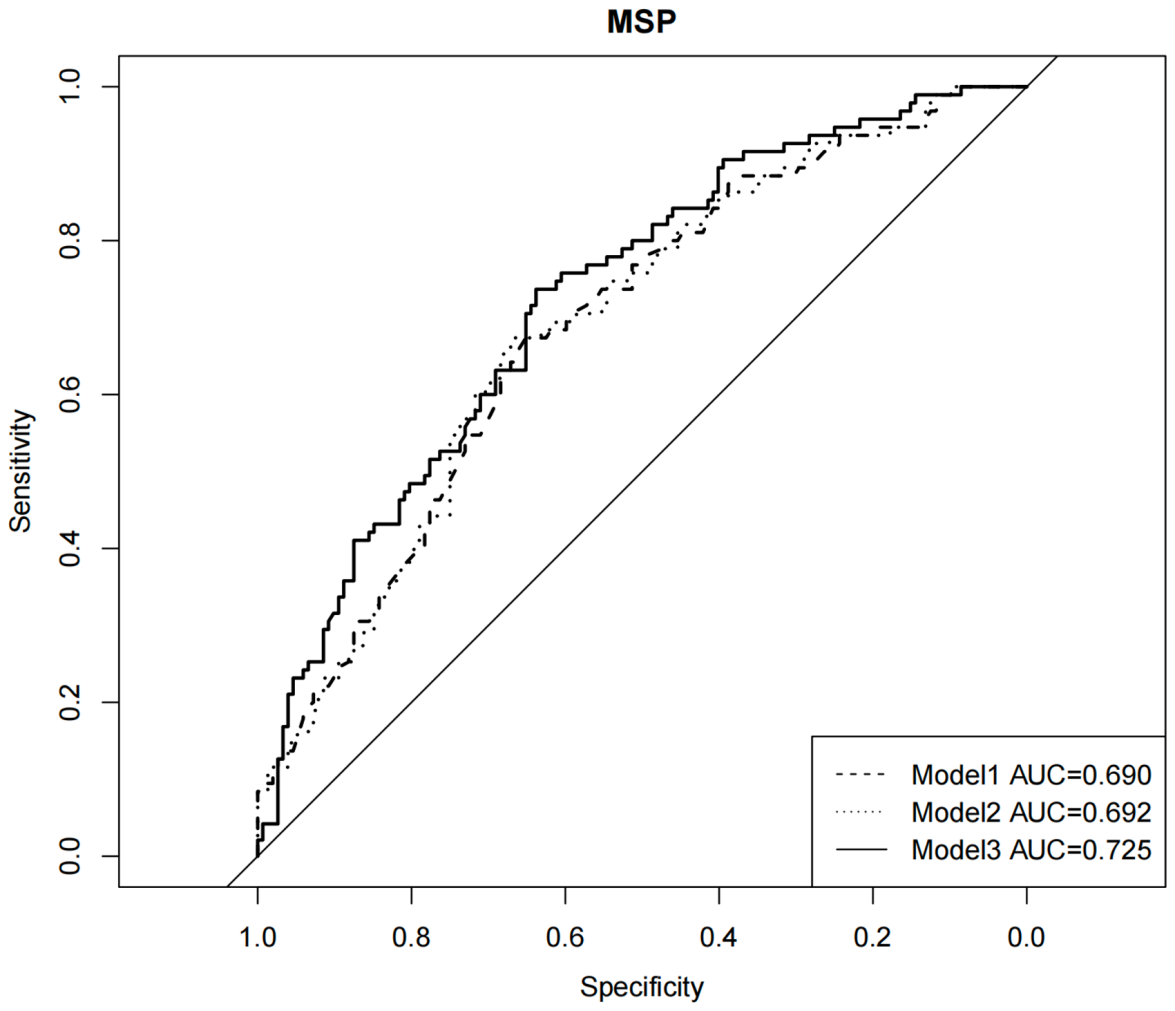
Receiver operator characteristic curve for demographic factors, HIV awareness variables, child sexual abuse in hierarchical logistic regression models predicting multiple sexual partners (*N*=247).

About 4 in 10 participants had anal sex with more than one male sexual partner in the past 6 months. When using hierarchical logistic regression on MSP, TGW with CSA history were at about three times higher risk of reporting MSP (*p* = 0.004, OR = 3.260; Table 3). Upon the addition of more variables, the models became more adaptable as demonstrated in Figure 2. All their AUC of them exceeded 0.6 and increased steadily.

## Discussion

Childhood sexual abuse is a worldwide public health issue that has not yet been resolved, and its impact on victims in adulthood has been the focus of research in psychology and behavioral medicine for nearly half a century. A high risk of HIV infection has emerged as a distinctive feature of those who have experienced CSA history, of which the mechanisms are still under-explored (Empson et al., 2017). TGW is a vulnerable group with high CSA prevalence in a dearth of targeted HIV control measures in most countries and territories, despite their disproportionate HIV burden (UNAIDS, 2022). To address this gap, our study first explored CSA exposure and its subsequent effects on sexual behavior among Chinese TGW, to raise awareness of sexual health in TGW and promote equal rights to health for people of all gender identities.

This study has several implications that are important to highlight. First, our findings have enriched the available data in the area of CSA impact and provided a novel perspective regarding Chinese TGW. Approximately 14.2% of participants in our survey reported CSA, which is in line with the previous results from college students in Asian countries (Menard and MacIntosh, 2021) but lower than the results from Chinese MSM (Jiang et al., 2020). It may be due to the fact that non-contact CSA has not been considered in this study. Social and historical causes of the comparatively low CSA exposure in Chinese society can both be identified. On the one hand, it is explicable that the actual number of CSA decreased with more nurturing care under the one-child policy (Tang et al., 2018). On the other hand, many victims do not realize that they have been violated, as they lack a sense of self-preservation and sexual consciousness. Influenced by Confucian culture, it is shameful to talk about sex openly in China, and children often have difficulty accessing appropriate sex education, for example, parents are embarrassed about responding to the sexual concerns raised by their children. Therefore, some participants who were involved in non-consensual sexual contact in childhood might reply “no” when they were asked whether had experienced CSA (Stoltenborgh et al., 2011; Zhu et al., 2015). In addition, TGW tends to avoid disclosures of CSA due to rare public recognition and the stigma it may attach to (Tang et al., 2018).

Second, it is noteworthy that the relationship between CSA and risky behaviors we found was consistent with traumagenic dynamics (TD) theory, which was one of the pioneers in searching for the adverse effect of CSA and has been validated among MSM, young females, or other vulnerable groups (Kalichman et al., 2004; Senn et al., 2012). TD theory argues that trauma results in a series of problem behaviors by altering the cognition and emotional orientation of a child, which subsequently distorts their self-concept, outlook, and emotional coping skills (Finkelhor and Browne, 1985). In other words, CSA victims have a poor understanding of sexual activities and confuse the boundaries of sexual morals, resulting in risky sexual behaviors. In this theory, four hypotheses are proposed as possible pathways of trauma, in which the traumatic sexualization and powerlessness are studied intensively (Matorin and Lynn, 1998). Traumatic sexualization refers to the inappropriate shaping of sexual behavior scripts that occur during CSA, leading victims to use sexuality to satisfy various purposes. For instance, CSA survivors engage in sexual relations with more than one partner for emotional response or material rewards. Alternatively, betrayal feelings caused by CSA could make it tough for victims to trust others and maintain intimacy, replaced by short-term sexual relationships. On the other hand, children will easily get disempowered as their willingness, desire, and sense of efficacy were inhibited when they were sexually assaulted. Persons with CSA may feel powerless in the sexual event afterward, which may lead to difficulties in sexual negotiation (Rotheram-Borus et al., 1996), including feeling unable to refuse safe intercourse and contributing to a lack of condom use (Senn et al., 2008; Walsh et al., 2013; Thornton and Veenema, 2015). In a word, our results suggest that the explanation given by TD theory is also applicable in Chinese TGW.

In our study, we also observed that stronger HIV risk perception appeared in TGW with CAI. The fear of contracting HIV possibly could be attributed to the risky sexual acts that have already taken place. No correlation between the response of HIV-KQ-18 and risky sexual behaviors was found. It is contrary to our conventional thinking that HIV knowledge represents a protective factor against engaging in risky behaviors. Similarly, research among young women had also not shown a correlation between HIV knowledge and condom use, and higher HIV knowledge was not associated with a reduction in the number of sexual partners in the cisgender population (Jalil et al., 2022). This highlights an important opportunity to improve HIV education, thus, it achieves its intended goals.

Evidence linking CSA to adverse sexual behaviors in our study has the following limitations. In particular, the absence of a probability sample prevents the generation of our results to the broader TGW population in China. Second, because of funding constraints, we could not access a more representative sample adjusted for inherent bias through respondent-driven sampling. However, the mode of recruitment we utilize is also one of the most commonly used in studies involving hidden populations (De Santis et al., 2010). Third, because of the mobility of TGW, the impact of survey sites has not been analyzed. Moreover, TGW without a history of anal intercourse in the past 6 months were excluded, which may bias the burden of CSA/risky sexual behaviors, as well as the relationship between them in this population. In addition, although there is temporal ordering between CSA and adult outcomes, a causal relationship cannot be established directly in this cross-sectional study. Another restriction in this article was the measurement of sexual abuse as a dichotomous variable, which underestimates the within-group heterogeneity of CSA victims (Senn et al., 2007; Boroughs et al., 2015), but it is easy to answer during an investigation.

There are several research directions we propose for the future. For instance, more characteristics of abuse should be collected and taken into account. The physical, emotional, and behavioral manifestations will vary from child to child, depending on the details of the abuse, which could inform treatment and prevention efforts. Furthermore, the role of age in initial victimization cannot be overlooked, because mental and neurological development can cause different responses to CSA (Rotheram-Borus et al., 1996). We also recommend further research to better describe the CSA effect on psychological activities, sexual behavioral consequences, and the mechanisms therein.

Moving forward, developing preventive policies is more economically efficient than taking remedial action, as the toll of disease or death in TGW survivors of CSA is quite considerable. On the one hand, cooperation and support from all sectors of society are necessary for practice. Obviously, forced sexual contact is calling for public attention, particularly the cases involving minors. The protective efforts to help victims of sexual violence should be improved by Chinese judicature and equal sexual rights could be promoted to safeguard sexual minorities from victimization (Wen, 2020). Second, this study calls for STI screening to be included in health promotion programs targeted for TGW (Reisner et al., 2016), and a reliable system should be established for collecting gender identity information in health records and providing TGW with HIV prevention services (CDC, 2021). Third, being surrounded by close friends and family can reverse the extreme behaviors of sexual abuse among survivors of abuse, and bolstering support from family members or peers may reduce vulnerability, enhance resilience, and improve positive coping strategies (Baytunca et al., 2017).

As WHO advocates, “No one person or discipline can effectively manage a case”; therefore (Djeddah et al., 2000), joint efforts from all disciplines are always recommended to reduce risky sexual behaviors among TGW in China.

## Conclusion

Among TGW in China, TGW with CSA were more prone to engage in CAI and have MSP. We identified that the occurrence of risky sexual behaviors has not shown a statistical association with HIV knowledge, but the HIV risk perception was a predictor for risky sexual behaviors. We also hope that our data reflect the importance of TGW voices in HIV policy development and advocacy to address childhood sexual abuse.

## Data availability statement

The datasets presented in this article are not readily available because the data generated by this study involves the privacy of the participants. Requests to access the datasets should be directed to YoC, caiyong202028@hotmail.com.

## Ethics statement

The studies involving human participants were reviewed and approved by Shanghai Jiao Tong University School of Medicine Public Health and Nursing Research Ethics Committee. The patients/participants provided their written informed consent to participate in this study.

## Author contributions

YiC: conceptualization, formal analysis, visualization, software, and writing–original draft. RC: data curation, validation, and writing–review and editing. FH: visualization, validation, software, and writing–review and editing. CX, XY, SL, DX, HC, RW, YL, and XG: data curation. TM: investigation. YW: writing–supervision, review and editing, methodology, and project administration. YoC: funding acquisition, project administration, conceptualization, methodology, and writing–review and editing. All authors have read and approved the final manuscript.

## Funding

This study was supported by the National Natural Science Funds of China under Grant [71673187] and the Shanghai Three-Year Action Plan for Public Health under Grants [GWV-10.2-XD13, GWV-10.1-XK15, and GWV-10.1-XK18]. The funding body had no role in the study design, collection, analysis, or interpretation of the data, writing the manuscript, or the decision to submit the article for publication.

## Conflict of interest

The authors declare that the research was conducted in the absence of any commercial or financial relationships that could be construed as a potential conflict of interest.

## Publisher’s note

All claims expressed in this article are solely those of the authors and do not necessarily represent those of their affiliated organizations, or those of the publisher, the editors and the reviewers. Any product that may be evaluated in this article, or claim that may be made by its manufacturer, is not guaranteed or endorsed by the publisher.

## Abbreviations

CSA: childhood sexual abuse
WHO: world health organization
TGW: transgender women
UNAIDS: the Joint United Nations Programme on HIV/AIDS
MSM: men who have sex with men
NGO: non-governmental organization
CI: confidence interval
INR: interquartile range
CAI: condomless anal intercourse
MSP: multiple sexual partners
HIV-KQ-18: HIV knowledge questionnaire 18 items
SD: standard deviation
K-S test: Kolmogorov–Smirnov test
ROC: receiver operating characteristic
AUC: area under the ROC curve
OR: odds ratio
REF: reference group
TD theory: traumagenic dynamics theory
