# Supplementary material for: Exploring the long-term sequelae of childhood sexual abuse on risky sexual behavior among Chinese transgender women
Source: Front Psychol. 2023 Apr 14;14:1057225. doi: 10.3389/fpsyg.2023.1057225 (PMC10140497; doi:10.3389/fpsyg.2023.1057225)
Supplement: Supplementary file 1 [file Table_1.doc]

**Supplementary Material**

**HIV Perceived Risk Scale (English Version)**

D11 Do you think you are a person who is vulnerable to HIV infection?

(1) Strongly agree (2) Agree (3) Don't know (4) Disagree (5) Strongly disagree

D12 I am worried that I may get AIDS

(1) Strongly agree (2) Agree (3) Don't know (4) Disagree (5) Strongly disagree

D13 How easy do you think it is for you to contract HIV?.

(1) very easy (2) easy (3) difficult (4) very difficult (5) never

D14 How worried do I feel about contracting AIDS?

1. very worried (2) very worried (3) moderately worried (4) a little worried (5) never worried

*The higher the score, the lower HIV risk perception.

**Original Edition in Chinese**

D11您觉得您是一个容易感染艾滋病的人吗？

（1）非常同意 （2）同意 （3）不知道 （4）不同意 （5）非常不同意

D12 我担心自己会感染艾滋病

（1）非常同意 （2）同意 （3）不知道 （4）不同意 （5）非常不同意

D13 您觉得自己感染艾滋病是：

（1）很容易 （2）容易 （3）困难 （4）很困难 （5）从来不会

D14 对于感染艾滋病这件事，我觉得的担心程度是：

非常担心 （2）很担心 （3）中度担心（4）一点点担心（5）从来不担心
